# Aloin isoforms (A and B) selectively inhibits proteolytic and deubiquitinating activity of papain like protease (PLpro) of SARS-CoV-2 in vitro

**DOI:** 10.1038/s41598-022-06104-y

**Published:** 2022-02-09

**Authors:** Devin S. M. Lewis, Joanna Ho, Savannah Wills, Anasha Kawall, Avini Sharma, Krishna Chavada, Maximilian C. C. J. C. Ebert, Stefania Evoli, Ajay Singh, Srujana Rayalam, Vicky Mody, Shashidharamurthy Taval

**Affiliations:** 1 Department of Pharmaceutical Sciences, School of Pharmacy, Philadelphia College of Osteopathic Medicine – Georgia Campus, Room 3031, 625 Old Peachtree Road, Suwanee, GA 30024 USA; 2 Chemical Computing Group, 910-1010 Sherbrooke W, Montreal, QC H3A 2R7 Canada; 3 Department of Pharmaceutical Sciences, South University, School of Pharmacy, Savannah, GA USA

**Keywords:** Diseases, Infectious diseases, Viral infection

## Abstract

The most common host entry point of human adapted coronaviruses (CoV) including SARS-CoV-2 is through the initial colonization in the nostril and mouth region which is responsible for spread of the infection. Most recent studies suggest that the commercially available oral and nasal rinse products are effective in inhibiting the viral replication. However, the anti-viral mechanism of the active ingredients present in the oral rinses have not been studied. In the present study, we have assessed in vitro enzymatic inhibitory activity of active ingredients in the oral mouth rinse products: aloin A and B, chlorhexidine, eucalyptol, hexetidine, menthol, triclosan, methyl salicylate, sodium fluoride and povidone, against two important proteases of SARS-CoV-2 PLpro and 3CLpro. Our results indicate only aloin A and B effectively inhibited proteolytic activity of PLpro with an IC_50_ of 13.16 and 16.08 μM. Interestingly, neither of the aloin isoforms inhibited 3CLpro enzymatic activity. Computational structural modelling of aloin A and B interaction with PLpro revealed that, both aloin isoforms form hydrogen bond with Tyr^268^ of PLpro, which is critical for their proteolytic activity. Furthermore, 100 ns molecular dynamics (MD) simulation studies predicted that both aloin isoforms have strong interaction with Glu^167^, which is required for PLpro deubiquitination activity. Our results from the in vitro deubiquitinase inhibition assay show that aloin A and B isomers exhibit deubiquitination inhibitory activity with an IC_50_ value of 15.68 and 17.51 µM, respectively. In conclusion, the isoforms of aloin inhibit both proteolytic and the deubiquitinating activity of SARS-CoV-2 PLpro, suggesting potential in inhibiting the replication of SARS-CoV-2 virus.

## Introduction

The outbreak of severe acute respiratory syndrome coronavirus 2 (SARS-CoV-2) resulting in coronavirus disease 19 (COVID-19) pandemic claimed millions of lives worldwide. SARS-CoV-2 is one of the β-CoVs and has single stranded RNA as a source of genetic material^1^. The virus's life cycle starts with spike proteins binding to the host cells' angiotensin-converting enzyme 2 (ACE2). Following the attachment, the viral envelop fuses with the host cell membrane, and the viral genome is released into the cytoplasm^2^. The virus's life cycle starts with the S1 subunit of spike (S) protein binding to the angiotensin converting enzyme 2 (ACE2) on the host cells. The S2 subunit of S protein anchors the S protein to the virion membrane and mediates the membrane fusion which is triggered by the cleavage of an additional site called the S2’ site. Two pathways for the entry of SARS-CoV-2 into host cells have been proposed upon the initial binding of the S1 subunit to ACE2. The binding of the S1 subunit to ACE2 exposes the S2’ site, which gets cleaved by transmembrane protease serine 2 (TMPRSS2) at the cell surface to bring the viral and cellular membranes together and create a fusion pore that allows the viral genome to reach the cell cytoplasm. If the target host cells express insufficient TMPRSS2, the entry of SARS-CoV-2 is mediated through the internalization of the virus–ACE2 complex via clathrin-mediated endocytosis^3^. The viral genome (ssRNA) uses the host ribosomes to translate the viral RNA into a long polypeptide chain (PP) of about 800 kDa. Two proteases encoded by the viral genome, papain like proteases (PLpro) and 3-chymotrypsin like protease (3CLpro), auto-cleave the newly formed PP chain to generate several non-structural proteins (NSPs) required for the viral replication. The PP chain is cleaved into 16 NSPs by PLpro and 3CLpro, with the 3CLpro generating 11 of the 16 NSPs making this protease the main target for developing anti-SARS-CoV-2 drugs^4^.

**Figure 1:**
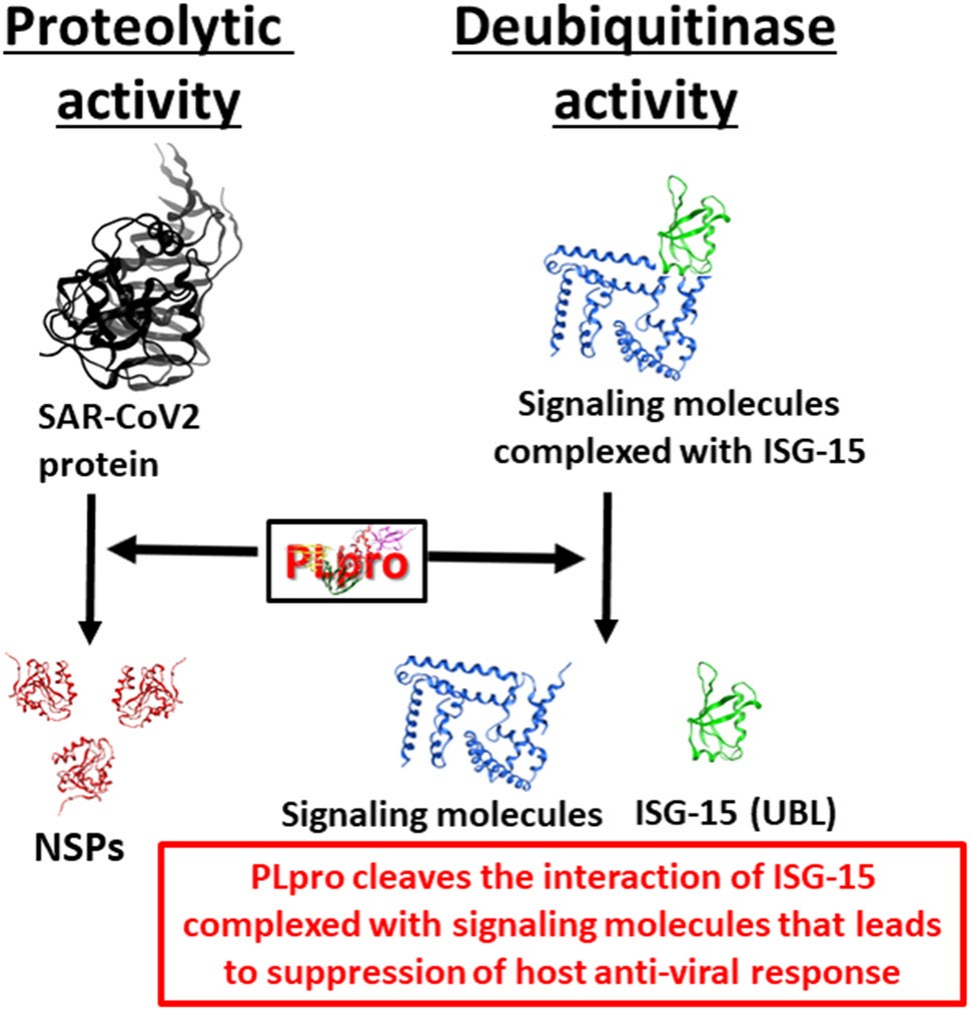
Schematic representation of proteolytic and DUB activity of PLpro. SARS-CoV-2 PLpro proteolyticaly cleaves the viral protein at three sites to generate the mature NSPs. Additionally, PLpro inhibits the binding of UBLs such as ISG-15 to signaling molecules that are required to elicit the type-I interferons mediated anti-viral immune response that leads to survival of the virus in the host and ultimately leading to viral-mediated pro-inflammatory cytokine response in host. *ISG* Interferon sensitive gene, *UBL* Ubiquitin like protein, *NSP* Nonstructural protein.

In addition to the protease activity, SARS-CoV-2 PLpro exhibits deubiquitination (DUB) activity^5^. Ubiquitination, a process of attachment of ubiquitin (UB) and ubiquitin like proteins (UBL) to the cellular proteins that needs to be degraded by the host proteasomal complex in cytosol, is an essential process required to maintain the host protein turn over. Ubiquitination also plays an important role in degrading the foreign proteins such as viral proteins upon infection to prevent the viral propagation^6^. Thus, the deubiquitination activity of SARS-CoV-2 PLpro leads to the disruption of host’s anti-viral immune response. During viral infection, innate immune cells such as dendritic cells produce Type-I interferon (IFN-α/β), which in turn activates interferon-sensitive gene-15 (ISG-15). The upregulated ISG-15 protein conjugates with various signaling molecules including JAK, STAT, IRF-3 through a process called ISGylation to mediate the Type-I IFN induced anti-viral function^7–9^. It has been shown that SARS-CoV-2 PLpro mediates deISGylation of ISG-15 to the host signaling molecules that leads to the inhibition of the host anti-viral innate immune response^7,10^. Therefore, the DUB activity of SARS-CoV-2 dysregulates the primary interferon mediated anti-viral response, which is the hallmark of COVID-19^11^. Plethora of reports suggest that the SARS-CoV-2 mediated mortality is by the pro-inflammatory cytokine storm^12^. The possible mechanism of pro-inflammatory cytokine storm upon SARS-CoV-2 infection might be due to the dysregulated interferon-mediated anti-viral response (Fig. 1). Thus, PLpro serves as a drug target not only to inhibit the viral replication but also to suppress the cytokine storm during SARS-CoV-2 infection. Taken together, these studies suggest that the drug candidates, which specifically inhibits the enzymatic activity of 3CLpro and PLpro will control the replication of SARS-CoV-2. However, the drugs that can specifically target DUB activity of SARS-CoV-2 PLpro may prevent the cytokine storm mediated tissue damage.

Although global vaccination is being currently undertaken, with new variants emerging, there are no SARS-CoV-2 specific FDA approved drugs to prevent the viral transmission from person to person. Several studies have shown that the major mode of transmission for SARS-CoV-2 is through respiratory droplets expelled from the infected person^13^. Additionally, the air droplet produced from asymptomatic individuals is one of the major silent source for the spread of the SARS-CoV-2 virus^14^. Importantly, the high initial viral loads are concentrated in mouth and nasopharyngeal region^15^. Therefore, it has become a huge risk factor for healthcare workers such as dentists, physicians, nurses who come in close contact with asymptomatic or infected patients. This warrants an urgent need to develop strategies to prevent the transmission of the virus. It has been shown that commercially available anti-septic mouthwash products exhibited anti-bacterial and anti-viral activity in the oral cavity^16^. After rinsing the mouth, Listerine and chlorhexidine reduced the herpes simplex virus-1 load in the saliva^17,18^. Recent studies have shown that several of the commercially available over the counter (OTC) mouthwash products are effective in inactivating the SARS-CoV-2 in an in vitro setting^19–21^. The mechanism could be due to the membrane-disruption or viral protein inactivation by the reagents used in the mouthwash^16^. However, the specific target of the active components used in the mouthwash to prevent the viral replication is still not clear.

Herein, we have investigated the inhibitory effect of active ingredients used in several commercially available mouthwash products through the 3CLpro and PLpro enzymatic activity assay. Most commonly found active ingredients in mouth rinses such as aloin A and B, chlorhexidine, eucalyptol, hexetidine, menthol, triclosan, methyl salicylate, sodium fluoride, and povidone iodide were studied for their inhibitory effects. We observed that none of these compounds were able to inhibit the 3CLpro enzymatic activity. However, both aloin A and B were able to inhibit more than 70% PLpro proteolytic and DUB activity. Our data suggest that aloin A and B might be potential drug candidates not only to inhibit the SARS-CoV-2 replication, but also to control the cytokine storm in COVID-19 patients. However, the SARS-CoV-2 anti-viral effect of aloin isomers need to be validated by preclinical and clinical studies.

## Results

### Inhibition of SARS-CoV-2 proteases by the active ingredients present in the mouthwash products

**Figure 2:**
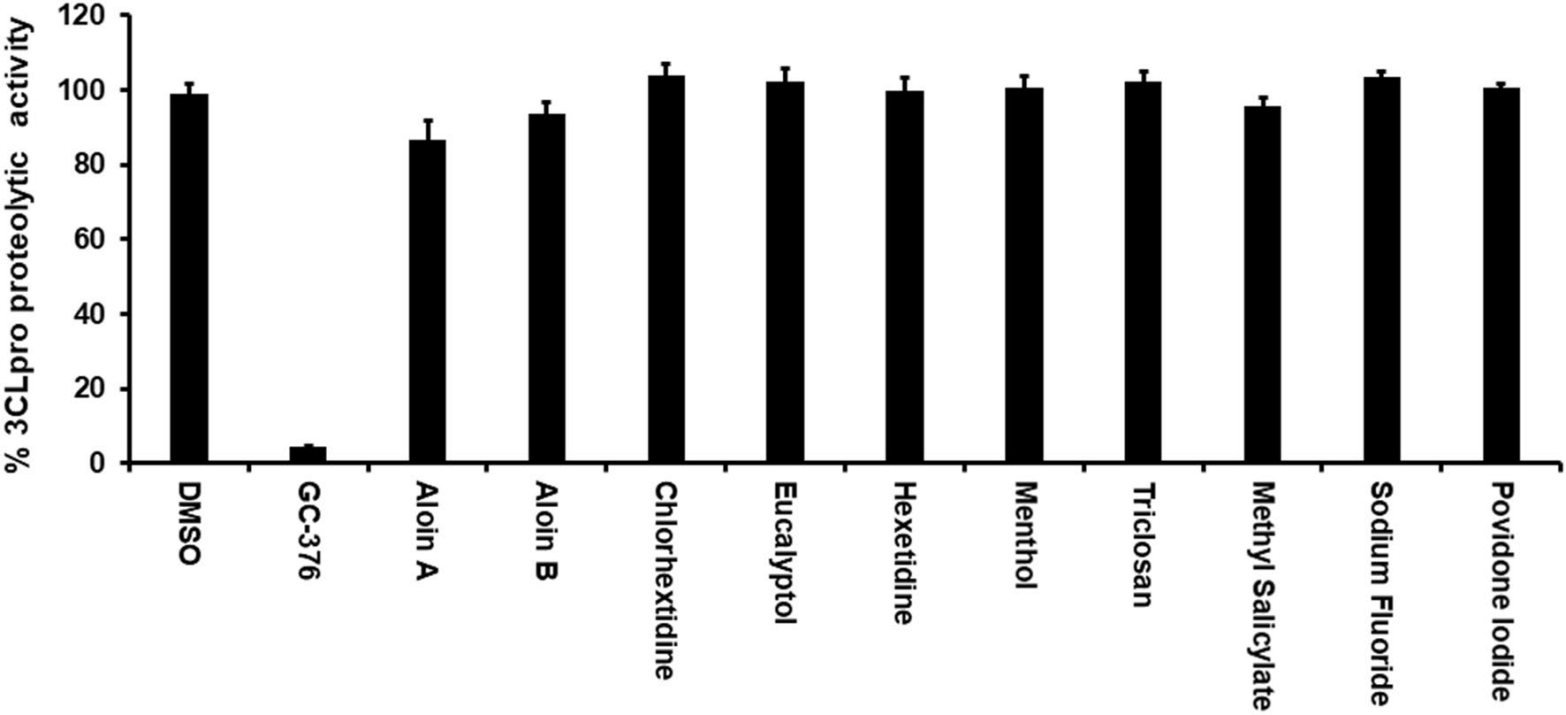
Most of the active ingredients present in the mouth rinses did not exhibit inhibitory activity against SARS-CoV-2 3CLpro enzyme. Aloin A, aloin B, chlorhextidine, ecalyptol, hexetidine, menthol, triclosan, methyl salicylate, sodium fluoride and providone iodide were selected for their inhibitory activity against SARS-CoV-2 3CLpro enzyme as described under Materials and Methods. The fluorescence intensity was used to calculate the percent enzymatic activity considering DMSO treated control as 100% activity. Blank values were subtracted from before calculating the percent activity. DMSO (0.1%) with enzyme and 50 µM of substrate served as positive control. Wells with 50 µM of GC-376 served as specificity controls for 3CLpro. Representative of three experiments (n = 3) with triplicate values were presented graphically. *P* value < 0.001 considered as statistically significant. One-way ANOVA with Bonferroni's Multiple Comparison post-hoc test was used to calculate the statistical significance.

**Figure 3:**
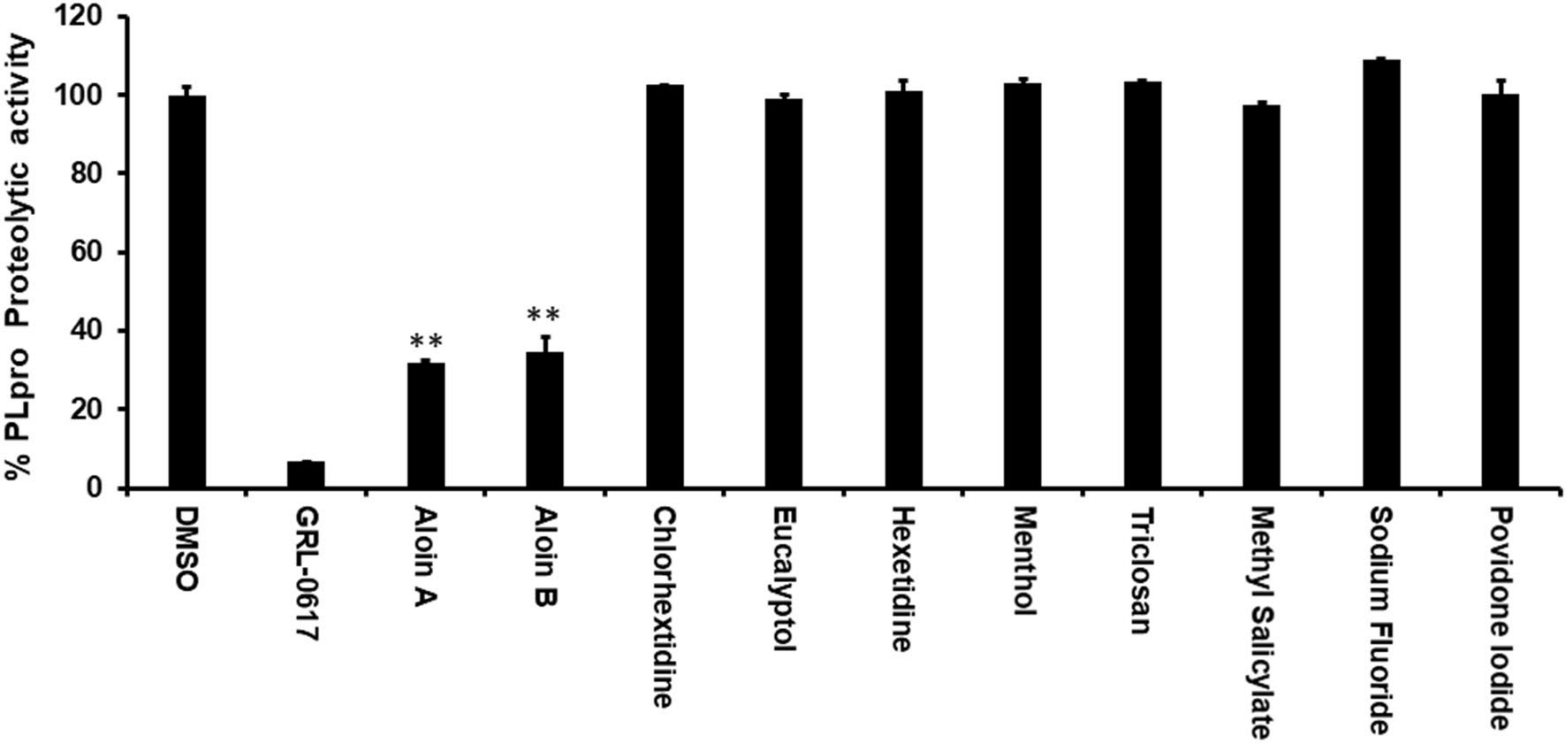
Aloin A and B specifically inhibits the SARS-CoV-2 PLpro proteolytic activity. Aloin A, aloin B, chlorhextidine, ecalyptol, hexetidine, menthol, triclosan, methyl salicylate, sodium fluoride and providone iodide were selected for their inhibitory activity against SARS-CoV-2 PLpro enzyme as described under Materials and Methods. The fluorescence intensity was used to calculate the percent enzymatic activity considering DMSO treated control as 100% activity. Blank values were subtracted before calculating the percent activity. DMSO (0.1%) with enzyme and 50 µM of substrate served as positive control. Wells with 50 µM of GR-L0617 compounds served as specificity controls for PLpro. Representative of three experiments (n = 3) with triplicate values were presented graphically. *P* value < 0.001 considered as statistically significant. One-way ANOVA with Bonferroni's Multiple Comparison post-hoc test was used to calculate the statistical significance.

Since commercially available mouth rinses are effective in inactivation of SARS-CoV-2^19–21^, we performed an in vitro enzymatic assay for the most important SARS-CoV-2 proteases such as 3CLpro and PLpro with active ingredients of mouth rinses. As shown in the Fig. 2, none of the active ingredients of the mouthwashes were able to inhibit the 3CLpro enzymatic activity at 50 µM concentration. Interestingly, as shown in Fig. 3, out of the 9 compounds tested, only aloin isomers (A and B from) were able to inhibit more than 70% of PLPro proteolytic activity at 50 µM concentration. These studies suggest that aloin isomers exhibit specific inhibitory activity towards SARS-CoV-2 PLpro.

### Structural interaction and 100 ns MD Simulation of aloin A and B with SARS-CoV-2 PLpro

The structure of SARS-CoV-2 PLpro is divided into four sub-domains, the N-terminal Ubiquitin-like domain, the α-helical Thumb domain, the β-stranded Finger domain and the Palm domain (Figure S1). This arrangement is similar to ubiquitin specific proteases deubiquitinating enzyme (DUB) with a very low homology (10%)^22^. The thumb comprises of six α helices and a small β hairpin. The fingers subdomain is made of six β strands and two α helices. The palm subdomain comprised of six β strands. It suggests that the proteolytic and DUB sites are independent of each other suggesting two possible activates of PLpro. The conventional catalytic triad Cys^111^-His^272^-Asp^286^ is located between the interface of palm and thumb subdomains. In addition to catalytic triad three additional residues play an important role in the enzymatic activity of SARS-CoV-2 PLpro: 1) An important β turn/loop (Glu^266^ -Gly^271^) which closes upon substrate and/or inhibitor binding is found adjacent to the active site. 2) Tyr^268^ part of the (Glu^266^ -Gly^271^) plays a critical role in proteolytic activity of SARS-CoV-2 PLpro. The mutation of Tyr^268^ has shown to interfere with the proteolytic activity of SARS-CoV-2 PLpro and hence any hydrogen bonding interaction of the inhibitor with Tyr^268^ will interfere with the proteolytic activity of the SARS-CoV-2 PLpro^22^. 3) Glu^167^ in SARS-CoV-2 PLpro plays an important role in ubiquitin core recognition, and mutations of Glu^167^causes a significant loss of DUB activity^22^. Similar to Tyr^268^, any hydrogen bonding interaction with Glu^167^ interferes with the DUB activity of the SARS-CoV-2 PLpro.

**Figure 4:**
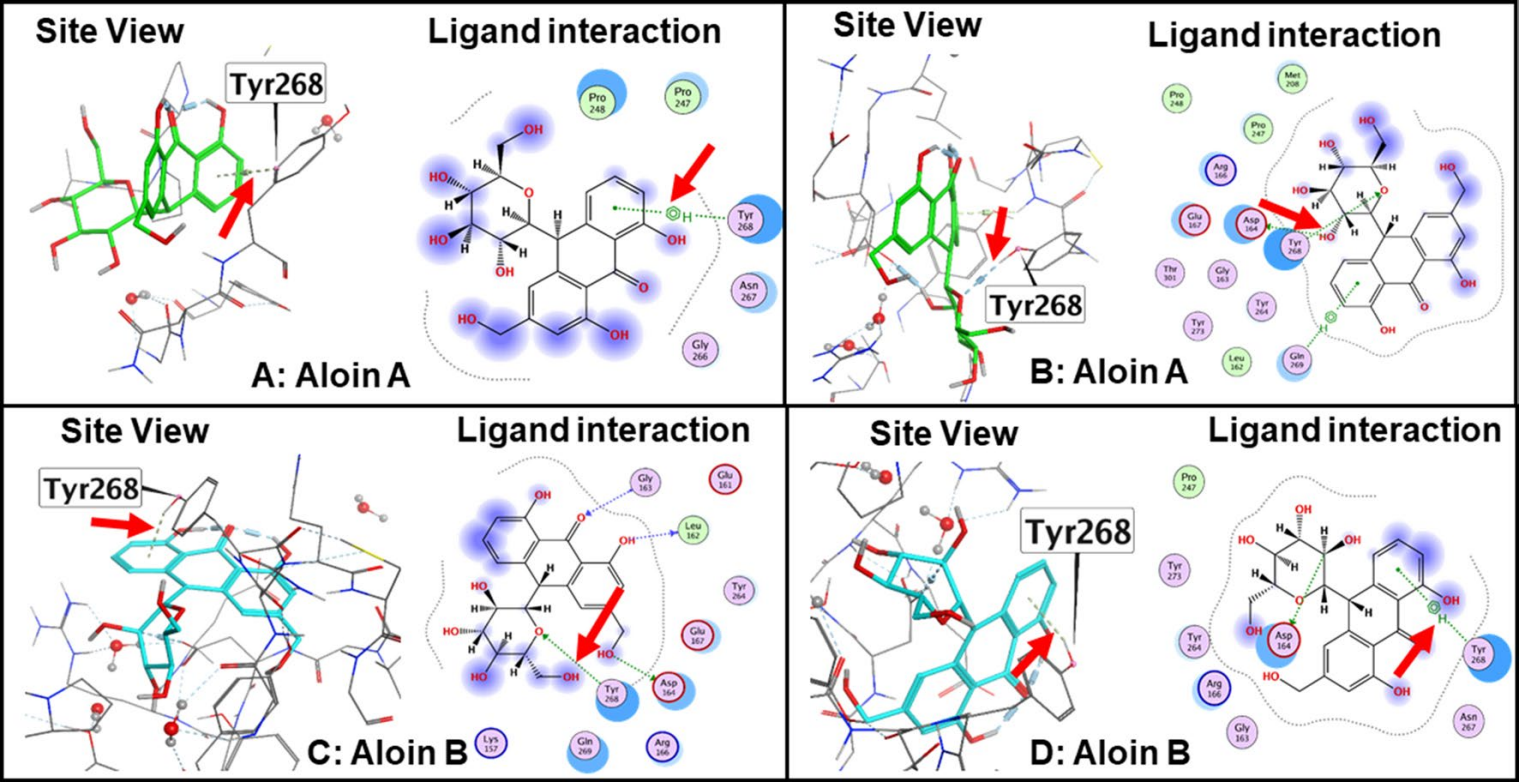
Structural analysis of interaction of aloin isomers with SARS-CoV-2 PLpro. (**A**) Interaction of aloin A orientation-1 with Tyr^268^ of PLpro through hydrogen bonding. (**B**) Interaction of aloin A orientation-2 with Tyr^268^ of PLpro through hydrogen bonding. (**C**) Interaction of aloin B orientation-1 with Tyr^268^ of PLpro through hydrogen bonding. (**D**) Interaction of aloin B orientation-2 with Tyr^268^ of PLpro through hydrogen bonding. Site view and ligand interaction maps were presented to illustrate the hydrogen bonding.

**Figure 5:**
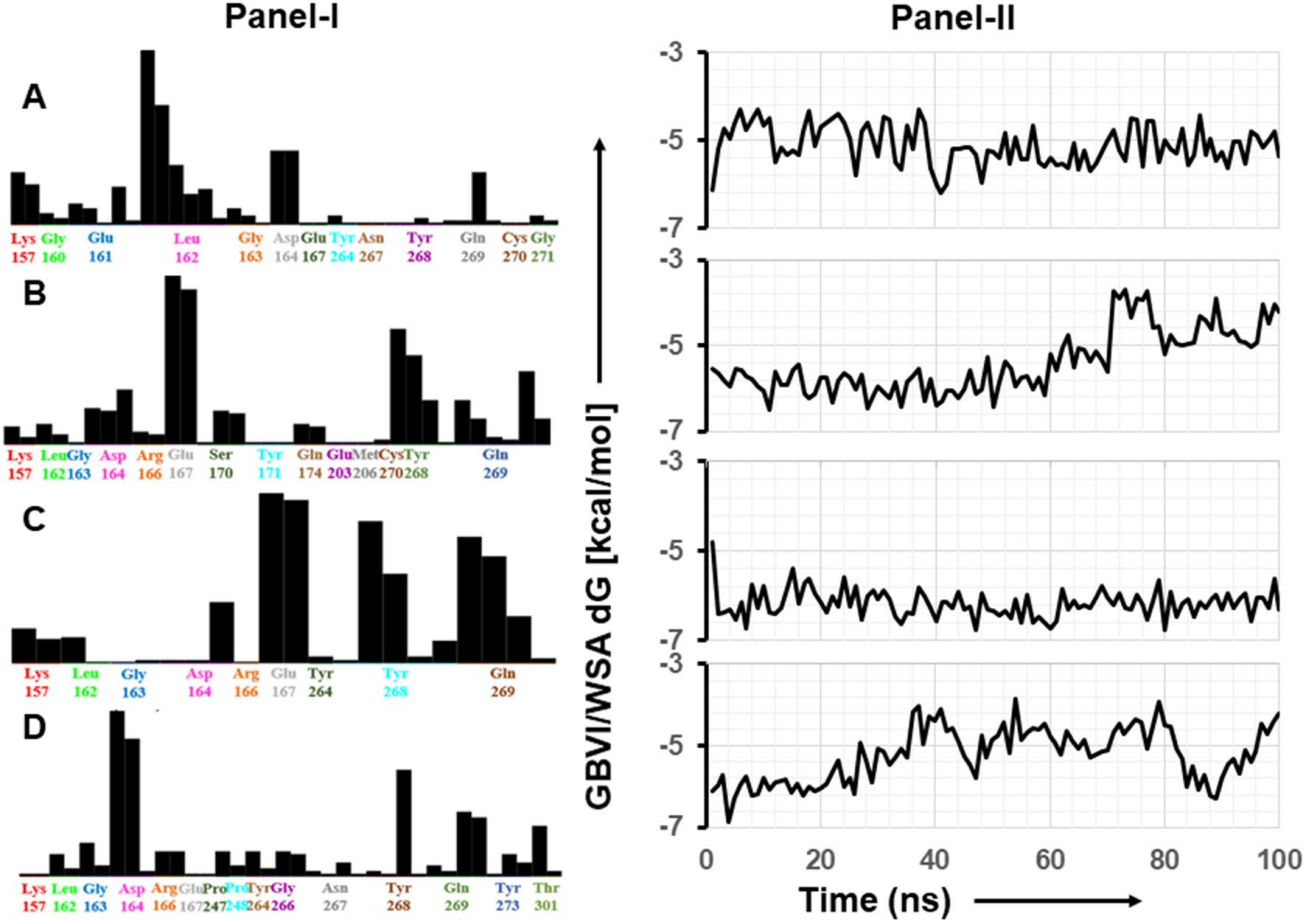
100ns MD simulation studies of aloin isomers with SARS-CoV-2 PLpro. The Fingerprint region and S-score of the two different orientations of aloin A and B with the SARS-CoV-2 PLpro enzyme. Fingerprinting map (Panel-I) and S-score distribution vs time scale (ns) (Panel-II) for aloin A and B with PLpro. (**A**) Aloin A orientation-1 shows weak interaction with Tyr^268^ and strong interaction with Glu^167^. (**B**) Aloin A orientation-2 shows interaction with Tyr^268^, Gln^269^ and Glu^167^. (**C**) Aloin B orientation-1 shows strong interaction with Try^268^, Gln^269^, and Glu^167^. (**D**) Aloin B orientation-2 shows strong interaction with Try^268^ and Gln^269^ but not with Glu^167^.

The interaction of aloin isomers to the ligand site of GRL0617, in the SARS-CoV-2 PLpro (PDBID: pbd7cmd) was analyzed using MOE software. Orientations that showed strong structural interaction with Tyr268 were considered for 100 ns MD simulation, as any hydrogen bonding interaction with Tyr^268^ will interfere with the proteolytic activity. Molecular docking studies of aloin A and B with PLpro resulted in 20 orientations for each of them. Four best orientations (two for each) with hydrogen bonding interaction at Tyr^268^ were chosen and then simulated with MD to evaluate the stability of these orientations at the 100ns time interval (Fig. 4A–D). The MD simulation data suggests that all four orientations were stable, and the molecules remained bound to the enzyme throughout the 100ns simulated time (Movies S1-S4). The analysis of protein–ligand interaction fingerprint between the SARS-CoV-2 PLpro enzyme and aloin A showed that the orientation-1 had a very weak Try^268^ interaction throughout the duration of simulation (Fig. 5A, panel-I). Orientation-1 for aloin A also showed significant interaction with Gln^269^ (Fig. 5A, panel-I). In contrast, orientation-2 for aloin A (Fig. 5B, Panel-I) showed significant interaction with Try^268^, Gln^269^, and Glu^167^. As mentioned earlier, Glu^167^ plays an important role in the deubiquitination of the enzyme and aloin A, molecular modeling predicted that the orientation-2 of aloin A significantly impairs the DUB activity of SARS-CoV-2 PLpro. The fingerprint region of aloin A with PLpro over 100ns time showed that the S-score for orientation-1 fluctuated from − 6.25 to − 4.25 kcal/mol but was stable for the period of computation (Fig. 5A, panel-II). The S-score for orientation-2 (− 6.5 to − 4.25 kcal/mol) of aloin A was stable for the first 80ns but fluctuated over the last 20ns of the calculation, however, the molecule always stayed bound to the enzyme over the period of study (Fig. 5B, panel-II). Hence based on the interaction of aloin A with Glu^167^, Tyr^268^, and Glu^269^, the orientation-2 of aloin A seems to be predominant for its interaction with the SARS-CoV-2 PLpro.

Figure 5C, D shows the fingerprint region and S-score of the two different orientations of aloin B with the SARS-CoV-2 PLpro enzyme. Figure 5C, panel-I shows that the orientation-1 of aloin B has a very strong interaction with Try^268^, Gln^269^, and Glu^167^ during the period of simulation similar to orientation-2 of aloin A (Fig. 5B, panel-I). These interactions of orientation-1 of aloin B with Try^268^, Gln^269^, and Glu^167^ can explain the strong inhibition of proteolytic as well as DUB activity of PLpro. The S-score for orientation-1 of aloin B ranged from − 6.75 to − 5.65 kcal/mol and was very stable over the period of 100ns (Fig. 5C, panel-II). Figure 5D, Panel-I shows that the orientation-2 of aloin B has a very strong interaction with Try^268^ and Gln^269^ but did not show any interaction with Glu^167^ during the period of simulation. The S-score for orientation-2 of aloin B was also very unstable throughout the period of simulation (Fig. 5D, panel-II) and varied from − 6.75 to − 3.85 kcal/mol. Hence based on the interaction of aloin B with Glu^167^, Tyr^268^, and Glu^269^ orientation-1 of aloin B seems to be predominant during its interaction with the SARS-CoV-2 PLpro. Thus, orientation-2 of aloin A and orientation-1 of aloin B with interactions with Glu^167^, Tyr^268^, and Glu^269^ can explain for their strong inhibition of both proteolytic and DUB activity of PLpro.

### Inhibition of deubiquitination (DUB) activity of SARS-CoV-2 PLpro by aloin isomers

**Figure 6:**
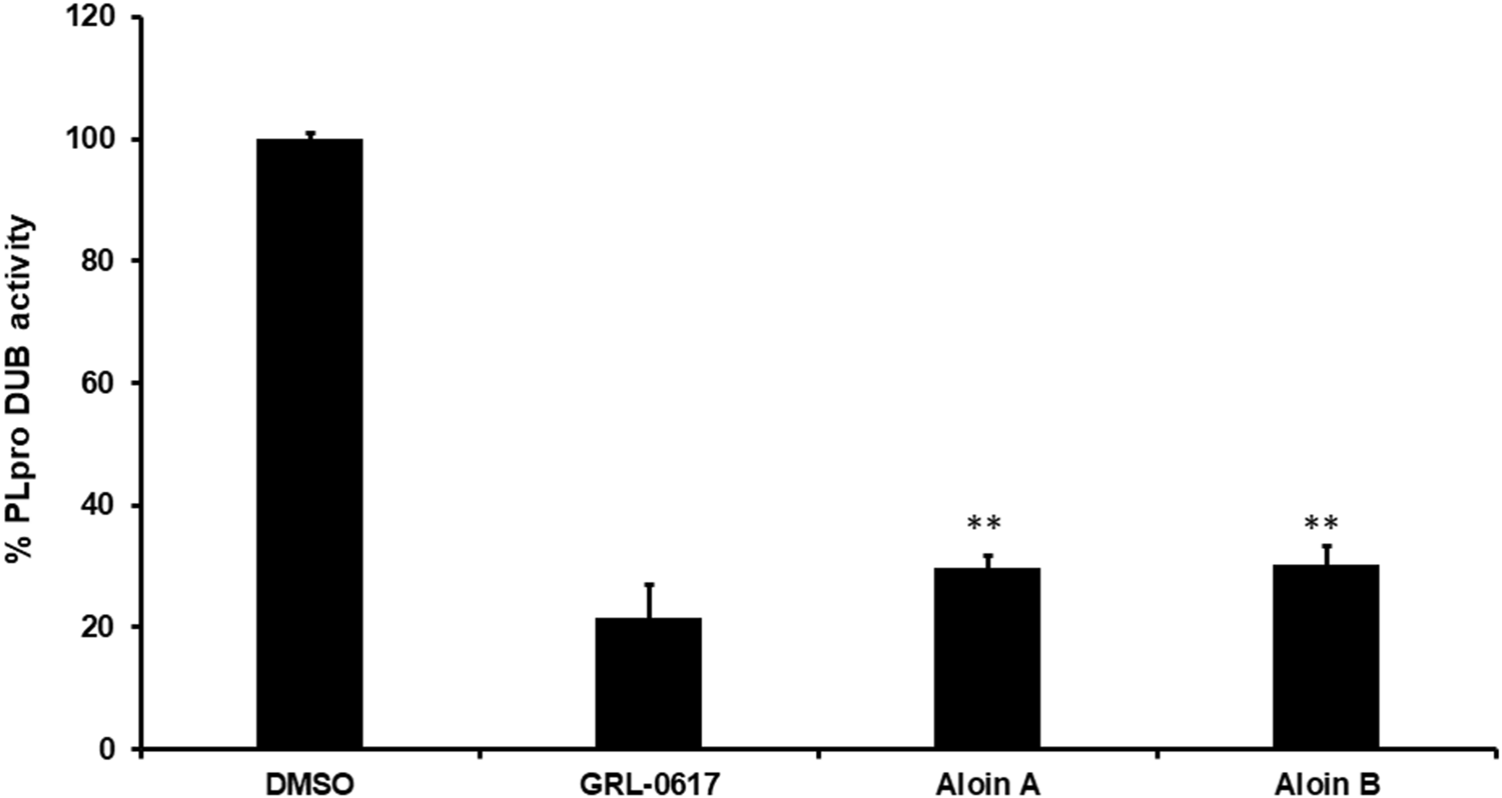
Aloin-A and B inhibits the SARS-CoV-2 PLpro DUB activity. Aloin A, and B isoforms were selected for their DUB inhibitory activity against SARS-CoV-2 PLpro enzyme as described under materials and methods. The fluorescence intensity was used to calculate the percent DUB activity considering DMSO treated control as 100% activity. Blank values were subtracted from before calculating the percent activity. DMSO (0.1%) with enzyme and 50µM of substrate served as positive control. Wells with 50µM of GRL-0617 compounds served as specificity controls for PLpro. Representative of three experiments (n = 3) with triplicate values were presented graphically. *P* value < 0.001 considered as statistically significant. One-way ANOVA with Bonferroni's Multiple Comparison post-hoc test was used to calculate the statistical significance.

Our MD Simulation data revealed that both aloin isomers may demonstrate DUB inhibitory activity of PLpro, therefore, we investigated the in vitro DUB activity of PLpro in the presence of aloin A and B. Previous data suggests that aloin isoforms inhibit the proteolytic activity of SARS-CoV-2 PLpro enzyme (Fig. 3). We performed the in vitro DUB activity using the Papain-like Protease (SARS-CoV-2) Deubiquitinase Assay Kit (BPS biosciences). As shown in Fig. 6, both the isoforms were able to inhibit more than 70% of DUB activity of the SARS-CoV-2 PLpro enzyme at 50µM concentration. These data aligns with the predicted interaction from MD simulation studies data suggesting that both aloin isomers A and B not only inhibits the proteolytic activity of SARS-CoV-2 PLpro but also its DUB activity.

### Dose and time dependent inhibition of both proteolytic and DUB activity of SARS-CoV-2 by aloin isomers

**Figure 7:**
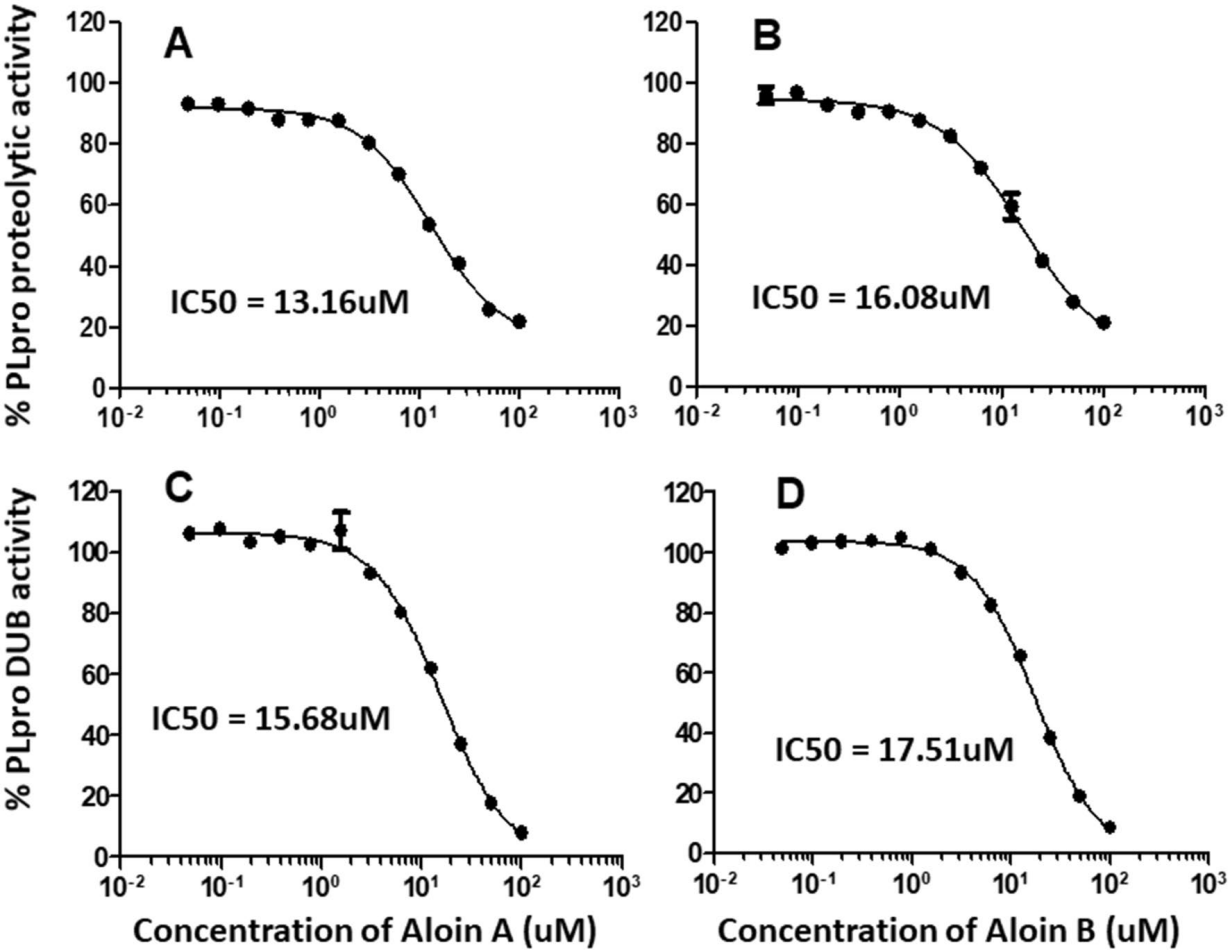
Dose-dependent inhibition of SARS-CoV-2 PLpro proteolytic and DUB activity by aloin A and B. A serial dilution of aloin A and B ranging from 0–100 µM in assay buffer was used. The percent activity was calculated as described in Fig. 2 legend. DMSO (0.1%) with enzyme and 50µM of substrate served as positive control. Wells with 50µM of GRL-0617 compounds served as specificity controls for PLpro. Representative of three individual experiments with triplicate values were presented graphically (n = 3). Non-linear regression (curve fit) with four variable dose vs inhibition was used to calculate the IC_50_ values using GraphPad Prism.

**Figure 8:**
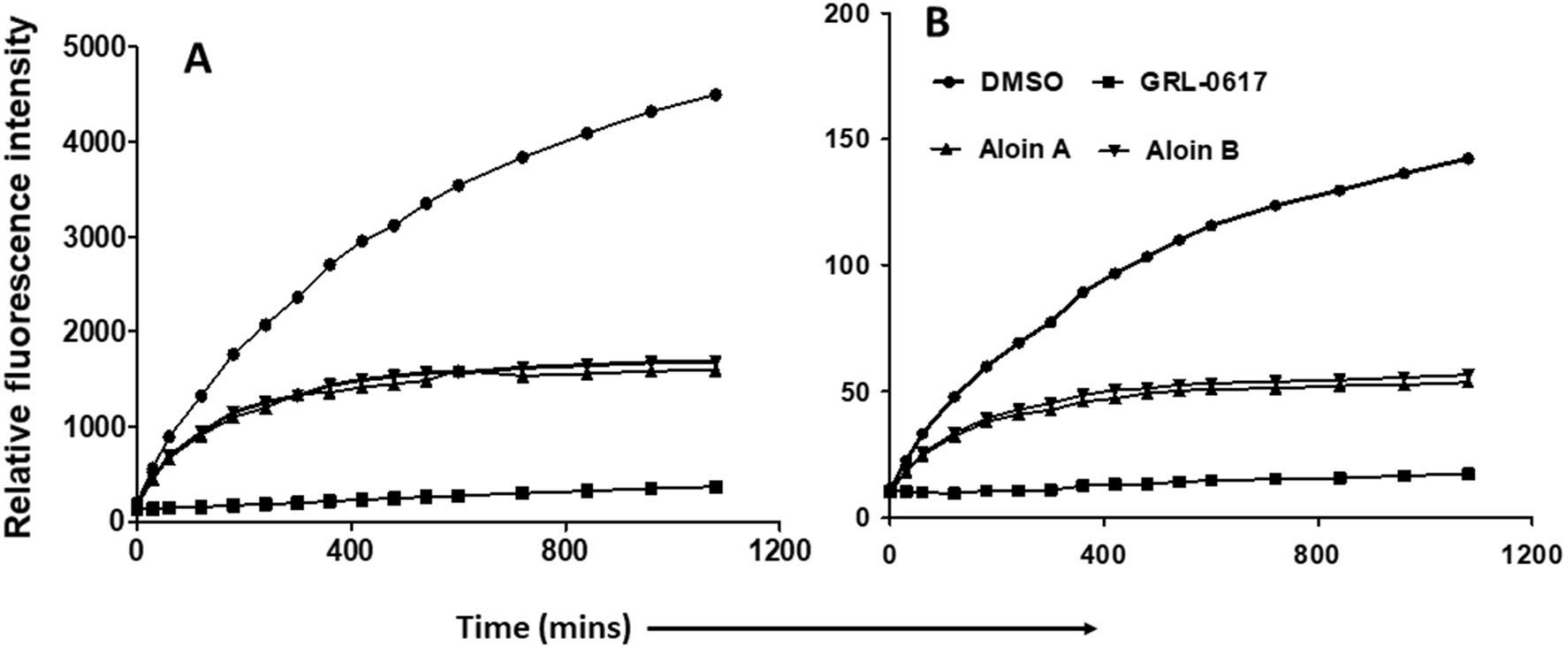
Time-dependent inhibition of SARS-CoV-2 PLpro proteolytic and DUB activity by aloin A and B. Aloin A and aloin B were tested for their time-dependent (0–18h) inhibitory activity at 50µM concentration against SARS-CoV-2 PLpro enzyme (**A**: proteolytic, **B**: DUB activity). The fluorescence intensity was used to calculate the percent enzymatic activity considering DMSO treated control as 100% activity. Blank values were subtracted before calculating the percent activity. DMSO (0.1%) with enzyme and 50µM of substrate served as positive control. Wells with 50µM of GRL0617 compounds served as specificity controls for PLpro. Representative of four experiments (n = 4) with triplicate values were presented graphically.

Next, we subjected both aloin A and B for further dose-dependent studies to calculate the concentration required to inhibit the 50% of PLpro enzymatic activity (IC_50_). IC_50_ is the most widely used measure of antagonist drug potency in pharmacological research. In this study, IC_50_ represents the concentration of aloin compounds required for 50% inhibition of PLpro and DUB enzymatic activity in vitro. As we observed in Fig. 7, aloin isomers inhibited around 80% proteolytic and DUB activity of PLpro at 100 µM concentration. The concentration of aloin isomers against the percent activity of PLpro was used to determine the IC_50_ with non-linear curve fit model as described in Methods section. The IC_50_ value for aloin A and B was found to be 13.16 and 16.08µM for proteolytic activity and 15.68 and 17.51µM for DUB activity, respectively. Further, the time dependent data (Fig. 8) suggests that the aloin isomers started exhibiting their inhibitory effect towards both proteolytic and DUB activity of PLpro as early as 1h and attained their maximum inhibitory effect by 4h under our assay conditions and the inhibition continued till 18h.

Additionally, aloin A and B did not exhibit cytotoxic effect on African green monkey kidney epithelial cells Vero-E6 (C1008) for 24 and 48h at 50 and 100µM concentration (Supplementary figure S2) respectively. Vero-E6 cells are known to be sensitive to SARS-CoV-2^23,24^ therefore we selected Vero-E6 cells for cell viability assay. Although aloin isomers did not alter the cell viability at the tested dose for up to 48h, it is possible that the aloin isomers cytotoxic effect may alter up on viral infection, therefore future studies are warranted for possible changes in the effective concentrations of aloin isomers.

Taken together, our data suggest that both aloin A and B are specific inhibitors of proteolytic and DUB activity of PLpro but not 3CLpro enzyme of SARS-CoV-2 virus and thus may help in the inhibition of SARS-CoV-2 viral replication.

## Discussion

SARS-CoV-2 virus is responsible for causing COVID-19, which is now a global threat especially with the emerging variants because of their high infectivity and mortality rate^25–28^. Though vaccines have been developed and are still in pursuit, there are no SARS-CoV-2 specific drugs available to stop the spread of this virus. Wearing mask is one of the means to prevent the spread of the virus; however, it is a challenge to control the spread of the virus in a setting where mask needs to be removed such as dental health care or encounters with asymptomatic individuals. SARS-CoV-2 mainly spreads through respiratory droplets^14^, therefore there is an immediate need to identify potential molecules that can reduce the viral load at the first point of entry such as the mouth and nasal region to prevent the spread of the virus . Recent studies have shown that most of the commercially available mouth rinses eliminate the SARS-CoV-2 virus in the mouth cavity by disrupting the outer envelope of the virus mainly because of the presence of peroxides or alcohol but these molecules also disrupt the host cells in the mouth cavity^16^. Therefore, it would be ideal to prepare mouthwashes with active ingredients that directly target the virus replication.

SARS-CoV-2 PLpro enzyme is a protease required to generate the NSPs essential for viral replication through its protease activity^29^. In addition, PLpro also exhibits de-ubiquitination and de-SIGylation in order to prevent the INF-α/β mediated anti-viral activity, thus may be responsible for pro-inflammatory cytokine storm in COVID-19 patients^5,30–32^. Therefore, PLpro serves as an excellent drug target not only to control the viral replication but also to prevent cytokine tsunami in COVID-19 patients. In this report, we have established that the aloin A and B inhibits the PLpro enzymatic activity. Aloin is an anthraquinone abundantly found in aloe vera plant. Anthraquinones including aloin A and B exhibit analgesic, antimicrobial^33–35^ and antiviral properties^36–38^. Recent reports suggest that aloin B inhibits hepatitis B virus replication in an in vitro setting^39^. Additionally, aloin exhibits anti-influenza activity by inhibiting the neuraminidase enzymatic activity including the oseltamivir-resistant influenza strain^40^. Further, pre-clinical studies suggest that aloin also promotes host immunity by enhancing the hemagglutinin specific T cells during PR8H1N1influenza infection in mice^40^. To the best of our knowledge, this is the first study to report that both aloin A and B specifically inhibit the SARS-CoV-2 PLpro proteolytic and DUB activity but not 3CLpro proteolytic activity. Both aloin A and B were able to inhibit more than 70% PLpro proteolytic activity. Structural analysis of aloin A and B with PLpro using computational studies revealed that both aloin isoforms form hydrogen bond with Tyr^268^ of PLpro, which is critical for their proteolytic activity. In addition, the 100ns MD simulation fingerprint analysis predicted a strong interaction of aloin A and B with Glu^167^, which is required for deubiquitination activity of PLpro. The in vitro experiments confirmed that both aloin A and B inhibited viral deubiquitination activity of PLpro enzyme suggesting their potential benefit in preserving anti-viral immune response, as well as, preventing the replication of SARS-CoV-2 virus. Mechanistically, our computational modeling studies suggest that aloin A and B interacts with the Glu^167^, Tyr^268^ and Glu^269^ residue of PLpro, which are essential for proteolytic and DUB activity, however, these studies need to be further validated by crystallographic data. Since PLpro enzyme is known to suppress the IFN-α/β mediated anti-viral response though DUB activity and responsible for cytokine storm in COVID-19 patients, use of aloin isoforms as an anti-SARS-CoV-2 drug may promote the IFN-α/β mediated anti-viral response and limit the cytokine storm in COVID-19 patients. In addition, it has also been shown that 1 ml of the aloe vera juice has approximately 10 μg/mL of aloin, and no cytotoxic effects were observed in vitro at the concentration of 120 μM of aloin. Furthermore, the recommended concentration of aloin for human consumption is 11mM^41^. Taken together, these studies suggest anti-viral effects of aloin from aloe vera with suitable safety profile.

In conclusion, several active ingredients present in most of the commercially available mouth rinses were selected as targets to screen the enzymatic inhibitory effect of SARS-CoV-2 specific 3CLpro and PLpro enzymes. Among the 10 active ingredients tested, only aloin A and B inhibited both PLpro proteolytic and DUB activities but not 3CLpro, suggesting the specificity of aloin A and B towards PLpro. Taken together, our data suggest that aloin A and B might be potential drug candidates not only to inhibit the SARS-CoV-2 replication, but also to control the cytokine storm in COVID-19 patients. However, the therapeutic potential of aloin A and B to prevent the spread of SARS-CoV-2 infection needs to be further validated by viral challenge and clinical studies.

## Materials and methods

### Reagents and drugs

Molecular biology grade DMSO was from Sigma Aldrich (St. Louis, MO, USA), Sterile PBS, penicillin/streptomycin (Pen/Strep) and PrestoBlue™ Cell Viability Reagent purchased from ThermoFisher Scientific (Waltham, MA, USA). African green monkey kidney epithelial cells Vero-E6 (C1008) cells and Eagle’s minimum essential medium (EMEM) were purchased from ATCC (Manassas, VA, USA). Fetal bovine serum (FBS) from Gemini Bio Products (Sacramento, CA, USA). Recombinant full length untagged 3CLpro, PLpro with His-Tag, assay buffers, inhibitors, fluorescently labelled substrates, Papain-like Protease (SARS-CoV-2), and deubiquitinase assay kits were from BPS Biosciences (San Diego, CA, USA). Aloin-A and B were purchased from MedChem Express. Chlorhexidine, eucalyptol, hexetidine, menthol, triclosan, methyl salicylate, sodium fluoride and povidone iodide were from (Sigma Aldrich). The names of the drugs, manufacturer name and catalog number are listed in Supplementary Table 1.

### Drug preparation

Eight milli molar stock solution of the compounds were prepared either in DMSO or PBS. Working solution of 250µM and 500µM of each compound was prepared in PBS and used for in vitro enzymatic assay.

### In vitro enzymatic assay

SARS-CoV-2 specific 3CLpro enzymatic assay was carried out as we previously reported^4^ and PLpro proteolytic assay was performed according to the manufacturer protocol. Briefly, 1ng/µl of 3CLpro and 0.4ng/µl of PLpro in 30µl of assay buffer was pre-incubated with the 10µl of 250µM compounds for 1h. Then, the enzymatic reaction was initiated by adding 10µl of 250µM fluorescently labeled substrate. Total volume of the assay samples was 50µl. Deubiquitinase activity of PLpro was estimated as per the manufacturer’s protocol (BPS Biosciences, San Diego, CA). The deubiquitinase assay conditions were similar to PLpro proteolytic assay but the reaction was initiated using PLpro specific ubiquitinated substrate (10µl of 25µM to make final concentration 1.25µM) and incubated for 16-18h at room temperature under dark condition. Fluorescent reading was taken at 360/40 excitation and 460/40nm emission using Synergy HT fluorescent plate reader. For dose-dependent studies, compounds were screened from 0-100µM range. 10µl of 1% DMSO with enzyme and 50µM of substrate served as positive control. For time-dependent studies 50µM of aloin A and B were incubated separately with PLpro and the enzymatic activity was monitored for 0-18h. Wells with 50µM of GC-376 and GRL-0617 compounds (BPS Biosciences) served as specificity controls for 3CLpro and PLpro respectively. Wells with 1% DMSO, 50µM of substrate and without enzyme served as blank. All the values were subtracted from blank values to calculate the percent activity of the enzymes.

### Cytotoxicity assay

The cytotoxic effect of aloin A and B was performed with Vero-E6 using PrestoBlue™ Cell Viability kit. The assay was carried out as per the manufacturer’s protocol. Briefly, around 20,000 cells were seeded for 6h in 96 well plates. Then the cells were refreshed with 100µl of media (EMEM with 10%FBS and 1% of Pen/Strep) along with 50 and 100µM aloin A and B and incubated for 24 and 48h at 37°C respectively. PrestoBlue solution (10µl) was added and continued the incubation for 1h at 37°C. The absorbance was taken at 530/25 excitation and 590/35nm emission using Bio-Tek Synergy HT fluorescent plate reader.

### PLpro protein preparation for computational studies

Molecular Operating Environment (MOE) 2020.09 was used to conduct the in silico studies using the Amber10:EHT forcefield. The crystal structure of COVID-19 PLpro was retrieved from the protein data bank (www.rcsb.org) with PDB format (ID: 7CMD). The protein was prepared by using MOE QuickPrep application with default settings. Corrections of structural errors, addition of hydrogens, calculation of partial charges, 3D optimization of H-bond network through Protonate3D and deletion of water molecules further than 4.5 Å from the protein and a restrained minimization of the system were performed. Crystallographic water molecules were kept based on the estimated solvent effects in COVID-19 PLpro-aloin binding calculated using the MOE Solvent Analysis application.

### Preparation of aloin isoforms

Both aloin A and B were prepared separately using ChemDraw Professional, Version 10, Cambridge Soft. The ‘wash’ function in MOE was used to rebalance protonation states and regenerate aloin A and B 3D coordinates to their minimum energy conformations.

### Protein: drug docking studies

Integrated Computer-Aided Molecular design computing method MOE was used to dock both aloin A and B with PLpro. Each aloin was docked using the Triangle Matcher placement method, and refined using the induced fit protocol. The docked molecules were scored with the GBVI/WSA dG scoring function^42^. In all, 20 binding conformations were collected for each aloin A and B. Two binding conformations for each aloin were retained for further analysis based on the highest binding affinity and orientation in the binding pocket.

### MD simulation studies

MD simulations were used to test the stability of the inhibitor in presence of docked aloin A and B. The simulation cell and NAMD 2.14^43^ input files were generated using MOE. The protein/ligand complexes were embedded in a TIP3P water box with cubic periodic boundary conditions, keeping a distance of 10Å between the boundaries and the protein. The net charge of the protein was neutralized with 100mM NaCl. For energy minimization and MD simulations, the AMBER10:EHT force field was used and the electrostatic interactions were evaluated by the particle-mesh Ewald method. Each system was energy-minimized for 5000 steps using the Steepest Descent and Conjugate Gradient method. For equilibration the system was subjected to a 100ps simulation to gradually heating the system from 10 to 300K. Next, a 100ps NVT ensemble was generated at 300K followed by an NPT ensemble for 200 ps at 300K and 1 bar. Then, for each complex, a 100ns production trajectory was generated for further analysis. The trajectory analysis was done using scripts shared by the CCG support group.

### Identification of ligand-binding mode

The protein–ligand interaction fingerprints (PLIF) descriptors implemented in the MOE were used. Interactions are classified as hydrogen bonds, ionic interactions, and surface contacts according to the residues. The PLIF descriptors for all protein-bound aloin were generated with the default parameter set in MOE over the recorded MD trajectories.

### Statistical analysis and reproducibility

Statistical analysis was carried out using one-way analysis of variance (ANOVA) with Bonferroni's Multiple Comparison test with 99.9% confidence intervals and represented as the mean ± SEM. Two-way ANOVA was carried out with Bonferroni's post-test to compare the grouped data. P values < 0.001 and P < 0.05 were considered statistically significant. Non-linear regression (curve fit) with four variable dose vs inhibition was performed to calculate the IC_50_ values. GraphPad Prism (version 6.07; La Jolla, CA, USA) was used for statistical analysis. The experiments were performed a minimum of three times with triplicates for reproducibility. Authors performing the assay were blinded for the drugs being tested in the assay.

## Supplementary Information

Supplementary Video 1.

Supplementary Video 2.

Supplementary Video 3.

Supplementary Video 4.

Supplementary Information 1.

## Data Availability

Data information can be obtained from the corresponding author upon reasonable request.
