# Supplementary material for: Aloin isoforms (A and B) selectively inhibits proteolytic and deubiquitinating activity of papain like protease (PLpro) of SARS-CoV-2 in vitro
Source: Sci Rep. 2022 Feb 9;12:2145. doi: 10.1038/s41598-022-06104-y (PMC8828865; doi:10.1038/s41598-022-06104-y)
Supplement: Supplementary file 5 — Supplementary Information 1. [file 41598_2022_6104_MOESM5_ESM.docx]

**Aloin isoforms (A and B) selectively inhibits proteolytic and deubiquitinating activity of papain like protease (PLpro) of SARS-CoV-2 *in vitro*.**

Devin Lewis^1#^, Joanna Ho^1#^, Savannah Wills^1#^, Anasha Kawall, Avini Sharma, Krishna Chavada, Maximillian Elbert^2^, Stefania Evoli^2^, Ajay Singh^3^, Srujana Rayalam^1^, Vicky Mody^1^*, Shashidharamurthy Taval^1^*

**^#^**Contributed equally

^1^Department of Pharmaceutical Sciences, School of Pharmacy, Philadelphia College of Osteopathic Medicine – Georgia Campus, Suwanee, GA, USA.

^2^ Chemical Computing Group, 910-1010 Sherbrooke W, Montreal, Canada, QC H3A 2R7.

^3^Department of Pharmaceutical Sciences, South University, School of Pharmacy, Savannah, GA, USA.

**Running Title:** SARS-CoV-2 PLpro inhibition by Aloin-A and B

**Key words:** SARS-CoV-2, 3CLpro, PLPro, mouth rinse, natural compounds, Aloin

***Corresponding Authors:**

Vicky Mody, Ph.D.,

Associate Professor, Department of Pharmaceutical Sciences,

Philadelphia College of Osteopathic Medicine, School of Pharmacy,

Room 3031, 625 Old Peachtree Road, Suwanee, GA-30024,

Tel: 678-407-7373, Fax: 678-407-7347, Email: [rangaiahsh@pcom.edu](mailto:rangaiahsh@pcom.edu)

Shashidharamurthy Taval, Ph.D.,

Associate Professor, Department of Pharmaceutical Sciences,

Philadelphia College of Osteopathic Medicine, School of Pharmacy,

Room 3031, 625 Old Peachtree Road, Suwanee, GA-30024,

Tel: 678-407-7373, Fax: 678-407-7347, Email: [rangaiahsh@pcom.edu](mailto:rangaiahsh@pcom.edu)

**Figure S-1.**


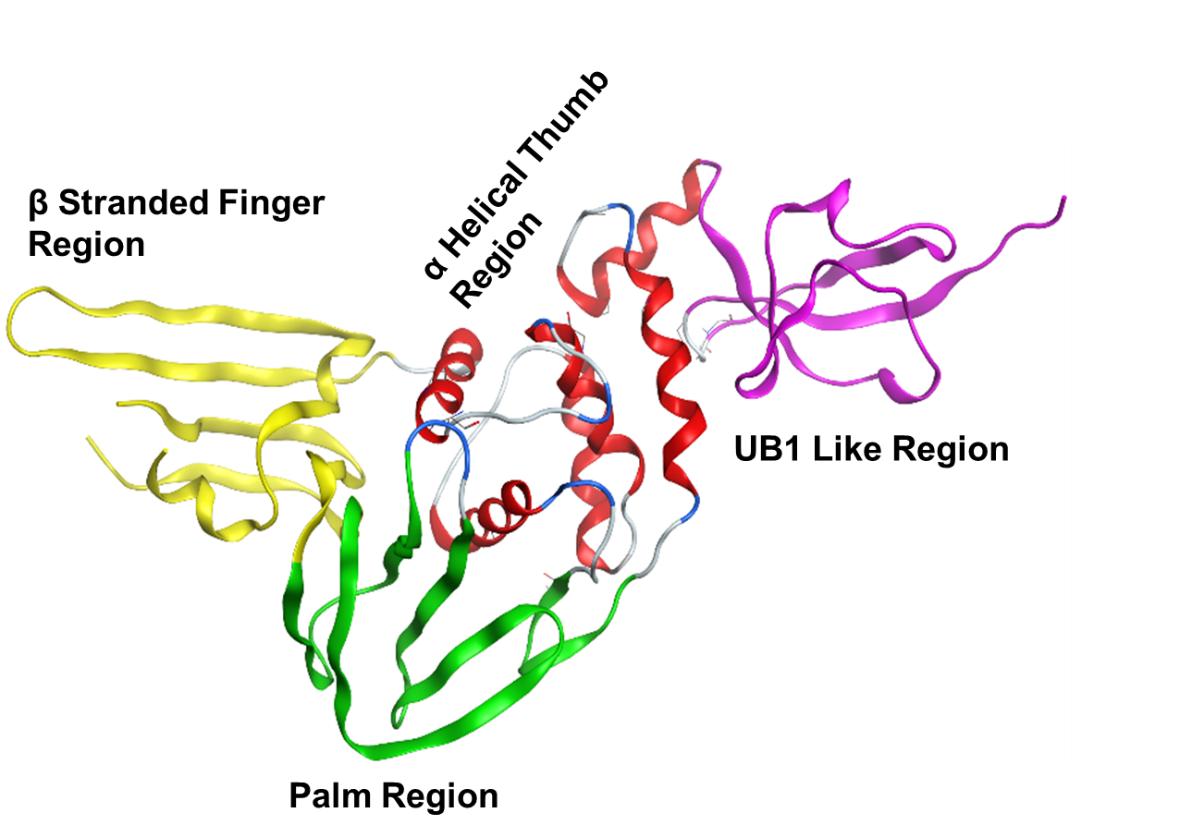


**Structure SARS-CoV2 PLpro enzyme.** The structure of SARS-CoV-2 PLpro is divided into four sub-domains, the N-terminal Ubiquitin-like domain (purple), the α -helical Thumb domain (red), the β -stranded Finger domain (yellow) and the Palm domain (green). The thumb comprises of six α helices and a small β hairpin. The fingers subdomain is made of six β strands and two α helices. The palm subdomain comprised of six β strands.

**Figure S-2.**


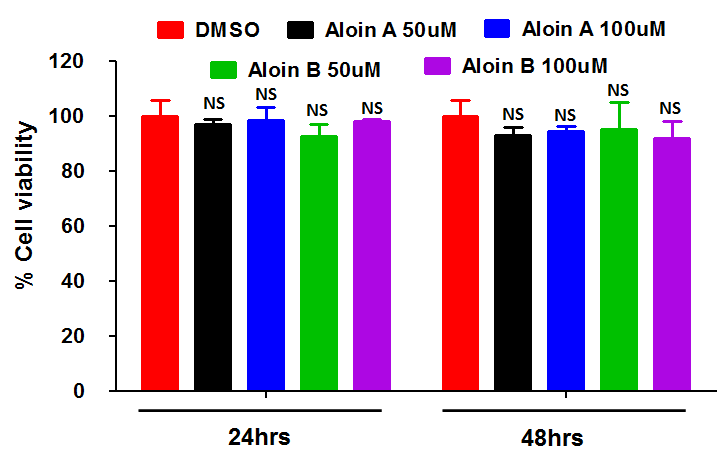


**Cytotoxic effects of aloin A and B in African green monkey kidney epithelial cell line Vero-E6.** Cytotoxicity assay was carried out using PrestoBlue™ Cell Viability kit as described under materials and methods. Both Aloin A and B were not cytotoxic to Vero-E6 cells at the tested doses up to 48h. Cells treated with 0.1% DMSO served as positive control. Representative of two individual experiments (n=2) with triplicate values were presented graphically. Two-way ANOVA with Bonferroni's Comparison post-test was used to calculate the statistical significance compare the DMSO treated cells with Aloin isomers treated cells. NS= Statistically not significant.

**Movie S-1.** MD simulation from 0-100ns for aloin A orientation 1 with PLpro.

**Movie S-2.** MD simulation from 0-100ns for aloin A orientation 2 with PLpro.

**Movie S-3.** MD simulation from 0-100ns for aloin B orientation 1 with PLpro.

**Movie S-4.** MD simulation from 0-100ns for aloin B orientation 2 with PLpro.

**Supplementary table 1:** List of active ingredient name, manufacturers and Catlog number

| **Drug** | **Manufacturer** | **Catalog Number** |
| --- | --- | --- |
| Aloin b | MedChem Express | HY-N0886 |
| Chlorhexidine | Cayman Chemicals | 26924 |
| Eucalyptol | Cayman Chemicals | 22183 |
| Hexetidine | MedChem Express | HY-B0996 |
| Menthol | Cayman Chemicals | 30384 |
| Triclosan | Cayman Chemicals | 20342 |
| Methyl salicylate | Sigma-Aldrich | M6752 |
| Sodium fluoride | Sigma-Aldrich | 201154 |
| Povidone iodide | Cayman Chemicals | 27883 |
